# SPOP promotes ATF2 ubiquitination and degradation to suppress prostate cancer progression

**DOI:** 10.1186/s13046-018-0809-0

**Published:** 2018-07-11

**Authors:** Jian Ma, Kun Chang, Jingtao Peng, Qing Shi, Hualei Gan, Kun Gao, Kai Feng, Fujiang Xu, Hailiang Zhang, Bo Dai, Yao Zhu, Guohai Shi, Yijun Shen, Yiping Zhu, Xiaojian Qin, Yao Li, Pingzhao Zhang, Dingwei Ye, Chenji Wang

**Affiliations:** 1 Department of Urology, Fudan University Shanghai Cancer Center, Shanghai, 200032 China; 2 State Key Laboratory of Genetic Engineering, Collaborative Innovation Center for Genetics and Development, School of Life Sciences, Fudan University, Shanghai, 200433 China; 3 Department of Oncology, Shanghai Medical College, Fudan University, Shanghai, 200032 China; 4 Department of Urology, Union Hospital, Tongji Medical College, Huazhong University of Science and Technology, Hubei, 430022 China; 5 Clinical and Translational Research Center, Shanghai First Maternity and Infant Hospital, Tongji University School of Medicine, Shanghai, 200040 China

**Keywords:** Prostate cancer, SPOP, ATF2, Ubiquitination, Proteasomal degradation

## Abstract

**Background:**

Next-generation sequencing of the exome and genome of prostate cancers has identified numerous genetic alterations. *SPOP* (Speckle-type POZ Protein) is one of the most frequently mutated genes in primary prostate cancer, suggesting that *SPOP* may be a potential driver of prostate cancer. The aim of this work was to investigate how SPOP mutations contribute to prostate cancer development and progression.

**Methods:**

To identify molecular mediators of the tumor suppressive function of SPOP, we performed a yeast two-hybrid screen in a HeLa cDNA library using the full-length SPOP as bait. Immunoprecipitation and Western Blotting were used to analyze the interaction between SPOP and ATF2. Cell migration and invasion were determined by Transwell assays. Immunohistochemistry were used to analyze protein levels in patients’ tumor samples.

**Results:**

Here we identified ATF2 as a bona fide substrate of the SPOP-CUL3-RBX1 E3 ubiquitin ligase complex. SPOP recognizes multiple Ser/Thr (S/T)-rich degrons in ATF2 and triggers ATF2 degradation via the ubiquitin-proteasome pathway. Strikingly, prostate cancer-associated mutants of SPOP are defective in promoting ATF2 degradation in prostate cancer cells and contribute to facilitating prostate cancer cell proliferation, migration and invasion.

**Conclusion:**

SPOP promotes ATF2 ubiquitination and degradation, and ATF2 is an important mediator of SPOP inactivation-induced cell proliferation, migration and invasion.

**Electronic supplementary material:**

The online version of this article (10.1186/s13046-018-0809-0) contains supplementary material, which is available to authorized users.

## Background

Prostate cancer accounts for 19% of estimated new cancer cases in men and is the third primary cause of male cancer-related mortality after lung cancer and colon cancer in United States [1]. In recent years, systematic sequencing studies have revealed that recurrent somatic mutation is a key feature of prostate cancer [2, 3]. Notably, the most frequently mutated gene in prostate cancer is *SPOP* (speckle-type POZ protein), which encodes a substrate adaptor for the Cullin3 E3 ubiquitin ligase complex, with recurrent mutation in up to 15% of prostate cancers [2–5].

CULLIN-RING ligases (CRLs) are a family comprised of more than 200 multi-subunit ubiquitin ligase complexes. Human cells express seven different Cullins (CUL1, 2, 3, 4A, 4B, 5, and 7), and each nucleates a multisubunit ubiquitin ligase complex [6]. The CRL3 complex is composed of the scaffold CUL3, the RING protein RBX1, and a BTB (Bric-a-brac/Tramtrack/Broad complex) domain protein that acts as an adaptor for substrate binding. There are > 180 BTB proteins in the human [7]. SPOP is a structurally well-characterized BTB protein that interacts with substrates via the MATH domain at its N terminus and binds CUL3 through the BTB domain at its C terminus [8]. The identification of SPOP-targeted substrates, such as BET proteins [9–11], ERG [12, 13], androgen receptor (AR) [14, 15], steroid receptor coactivator 3 (SRC-3) [16], Cdc20 [17] and SENP7 [18], have revealed a role for SPOP in regulating multiple cellular processes, including androgen receptor-dependent signaling, epigenetic control, and cell cycle regulation. Notably, prostate cancer-associated SPOP mutants are deficient in binding and promoting the degradation of substrates, leading to increased prostate cancer cell proliferation and invasion [3, 14], indicating the loss of function of SPOP mutations and the tumor-suppressive role of SPOP in prostate cancer. The identification of additional SPOP substrates may help to elucidate the underlying molecular mechanisms of *SPOP*-mutated prostate cancer.

Activating Transcription Factor 2 (ATF2) is a member of the ATF/CREB bZIP family of transcription factors, which heterodimerizes with members of the JUN and FOS transcription factor families [19, 20]. ATF2 is a phosphorylation substrate of JNK and p38. In response to stress stimuli, these kinases phosphorylate ATF2 at two key threonine residues in the N-terminal transactivation domain (TAD), leading to its activation [21]. ATF2 activation led to upregulation of a variety of transcriptional targets including cyclin A, cyclin D and MMP-2, which are involved in oncogenesis in various tissue types [22]. ATF2 acts as an important oncogene in prostate cancer, melanoma, non-small cell lung carcinoma and pancreatic cancer [23–26], while ATF2 exhibits tumor suppressor functions in nonmalignant skin and breast cancer [23, 27], suggesting a context-dependent role for ATF2 in cancer biology. When ATF2 functions as an oncogene, its expression is associated with poor prognosis and metastatic burden, and a role for ATF2 in driving metastatic progression of these tumors has been suggested [20, 24, 25, 28–30].

In this study, we demonstrated that SPOP forms a functional CUL3-SPOP-RBX1 E3 ubiquitin ligase complex that targets ATF2 for ubiquitination and proteasomal degradation in prostate cancer cells, contributing to facilitating prostate cancer cell migration and invasion. Moreover, this effect is abrogated by prostate cancer-associated SPOP mutations. Our results provide a functional insight into the underlying molecular mechanism of prostate cancer with SPOP mutations.

## Materials

### Cell culture and transfection

293 T and prostate cancer cell lines C4–2 were obtained from the American Type Culture Collection (ATCC). 293 T and C4–2 cells were maintained in DMEM with 10% (*v*/v) FBS. All cells were grown at 37 °C with 5% CO_2_.

### Expression constructs

Expression vectors for SPOP-WT or mutants are described previously [31]. ATF2, CREB1, C-Fos and C-Jun cDNAs were amplified from 293 T cDNA library, and subcloned into PCMV-FLAG vector. ATF2 mutants were generated by KOD-Plus-Mutagenesis Kit (TOYOBO) following the manufacturer’s instructions. All the constructs were verified by DNA sequencing.

### Lentiviral preparation, viral infection, and stable cell generation

The pLKO.3G GFP-shRNA plasmids were purchased from Addgene. The shRNA sequence of sh-SPOP#1: 5’-GGAGAACGCUGCAGAAAUU-3′; sh-SPOP#2: 5’-ATAAGTCCAATAACGACAGGC-3′; shATF2-#1: 5′- GAAATCTGTGGTTGTAAAT -3′; shATF2-#2: 5’-ATCATTACAGGTTCCCAAT-3′; shControl: 5′- ACAGACUUCGGAGUACCUG-3′. Viruses were collected from the medium 48 h after transfection. For knockdown experiments, cells were infected with the collected viruses over 48 h in the presence of polybrene, followed by GFP sorting for 3–4 days.

### Immunoprecipitation

For immunoprecipitation of the FLAG-tagged proteins, transfected cells were lysed 24 h after transfection with BC100 buffer. The whole-cell lysates were immunoprecipitated by overnight incubation with monoclonal anti-FLAG antibody-conjugated M2 agarose beads (Sigma). After three washes with FLAG lysis buffer, followed by two washes with BC100 buffer, the bound proteins were eluted from the beads with FLAG-Peptide (Sigma)/BC100 and were subjected to Western blotting. For immunoprecipitation of the endogenous proteins, cells were lysed with cell lysis buffer (Cell Signaling), and the lysates were centrifuged. The supernatant was precleared with protein A/G beads (Sigma) and incubated with the indicated antibody overnight at 4 °C. The immunocomplexes were then incubated for 2 h at 4 °C with protein A/G beads. After centrifugation, the pellets were collected and washed five times with lysis buffer, resuspended in sample buffer, and further analyzed by SDS-PAGE.

### Western blotting

Cell lysates or immunoprecipitates were subjected to SDS-PAGE, and then proteins were transferred onto nitrocellulose membranes (GE Healthcare). The membranes were blocked in Tris-buffered saline (TBS; pH 7.4) containing 5% nonfat milk and 0.1% Tween-20, washed three times in TBS containing 0.1% Tween- 20, and incubated with the primary antibody overnight at 4 °C, followed by the secondary antibody for 1 h at room temperature. Antibody binding was visualized using the ECL Chemiluminescence System (Santa Cruz).

### Antibodies and chemicals

The following antibodies were used: SPOP (16750–1-AP; proteintech), ATF2 (ab32160; Abcam), DEK (16750–1-AP; proteintech), Myc (9E10; Sigma), FLAG (M2; Sigma), HA (MM5-101R; Convance), Actin (AC-74; Sigma).

### Quantitative RT-PCR

Total RNA was isolated from C-42 cells using the Trizol reagent (Invitrogen, USA), and cDNA was reversed-transcribed using the Superscript RT kit (TOYOBO, Japan) according to the manufacturer’s instructions. PCR amplification was performed using the SYBR Green PCR master mix Kit (TOYOBO, Japan). All quantization were normalized to the level of endogenous control *GAPDH*. The primer sequences for the qPCR used are as follows: *SPOP*-F: 5’-AGCAAATGATAAACTGAAAT-3′; *SPOP*-R: 5′- GTCATCAGGGAGAAGCCCGT-3′; *SOX9*-F: 5- ATGAAGATGACCGACGAGCA-3; *SOX9*-R: 5′- AAGGGCCGCTTCTCGCTCTC -3; *MMP9-*F: 5′- GAGTTCCCGGAGTGAGTTGA-3′; *MMP9-*R: 5′- AAAGGTGAGAAGAGAGGGCC-3′; *TGFB2*-F: 5′- CCCTAAGCGAGCAATTCCAC -3′; *TGFB2*-R: 5′- CTGCTCCTCCTTCTCTTGCT -3′.

### Cell proliferation assay

Cell proliferation rate was determined using Cell Counting Kit-8 (CCK-8) according to the manufacturer’s protocol (Dojindo Laboratories, Japan). Briefly, the cells were seeded onto 96-well plates at a density of 1000 cells per well. During a 2 to 8-d culture periods, 10 μl of the CCK-8 solution was added to cell culture, and incubated for 2 h. The resulting color was assayed at 450 nm using a microplate absorbance reader (Bio-Rad). Each assay was carried out in triplicate.

### Migration and invasion assays

Cell migration and invasion were determined by Transwell (Costar) migration and invasion assays. C4–2 cells were precultured in serum-free medium for 48 h. For migration assay, 3 × 10^4^ cells were seeded in serum-free medium in the upper chamber, and the lower chamber was filled with DMEM containing 10% FBS. After 48 h, the non-migrating cells on the upper chambers were carefully removed with a cotton swab, and migrated cells underside of the filter stained and counted in nine different fields. Matrigel invasion assays were performed using Transwell inserts (Costar) coated with Matrigel (BD Biosciences)/fibronectin ((BD Biosciences).

### Samples from individuals with prostate cancer

Treatment-naive prostate cancer samples were collected from the radical prostatectomy series at Fudan University Shanghai Cancer Center. H&E slides of frozen and formalin-fixed, paraffin-embedded (FFPE) human tumor tissues were examined by a general pathologist and a genitourinary pathologist to confirm histological diagnoses and Gleason score and to verify the high-density cancer foci (> 80%) of the selected tumor tissue. The frozen blocks for DNA extraction, followed by ten consecutive 10-μm sections of each tumor, were examined by the pathologists as described above. These qualified samples were then used for DNA isolation. FFPE tissues were used for IHC analyses.

### Detection of prostate cancer specimens with SPOP mutations by sanger sequencing

For Sanger sequencing, DNA was extracted from all 90 cases of FFPE prostate cancer tissue using a QIAamp DNA FFPE Tissue kit. PCR was performed using 2 × Hot Start Taq Master Mix from novoprotein, and PCR products were purified using a GeneJET Extraction kit according to the manufacturer’s instruction and used for Sanger sequencing. The primers used for DNA amplification were as follows: Amp-Exon6-Forward 5′-ACCCATAGCTTTGGTTTCTTCTCCC-3′; Amp-Exon6-Reverse 5′-TATCTGTTTTGGACAGGTGTTTGCG-3′; Amp-Exon7-Forward 5′-ACTCATCAGATCTGGGAACTGC-3′; Amp-Exon7-Reverse 5′-AGTTGTGGCTTTGATCTGGTT-3′. Amp-Exon6-Reverse and Amp-Exon7-Forward were also used for Sanger sequencing.

### Immunohistochemistry

FFPE tumor samples from patients were deparaffinized, rehydrated and subjected to heat-mediated antigen retrieval. The UltraSensitive S-P (Rabbit) IHC Kit (KIT-9706, Fuzhou Maixin Biotech) was used following the manufacturer’s instructions with minor modifications. Briefly, sections were incubated with 3% H2O2 for 15 min at room temperature to quench endogenous peroxidase activity. After antigen retrieval using unmasking solution (Vector Labs), slides were blocked with normal goat serum for 1 h and then incubated with primary antibody at 4 °C overnight. IHC analysis of tumor samples was performed using primary antibodies against ATF2 (dilution 1:100; Abcam, ab32160). The sections were then washed three times in 1× PBS and treated for 30 min with biotinylated goat-anti–rabbit IgG secondary antibodies (Fuzhou Maixin Biotech). After washing three times in 1× PBS, sections were incubated with streptavidin-conjugated HRP (Fuzhou Maixin Biotech). After washing three times in 1× PBS for 5 min each, specific detection was developed with 3,3′-diaminobenzidine (DAB-2031, Fuzhou Maixin Biotech). Images were acquired using an Olympus camera and matched software. IHC staining was scored by two independent pathologists on the basis of the ‘most common’ criteria.

## Results

### Identification of ATF2 as a novel SPOP interacting protein

To identify molecular mediators of the tumor suppressive function of SPOP, we performed a yeast two-hybrid screen in a HeLa cDNA library using the full-length SPOP as bait. A total of 23 SPOP interacting clones were obtained and 4 clones were corresponding to ATF2 fragments (Additional file 1: Table S1). Since the SPOP-ATF2 interaction has not been previously reported in the literature, we examined the potential functional relationship between SPOP and ATF2.

**Fig. 1:**
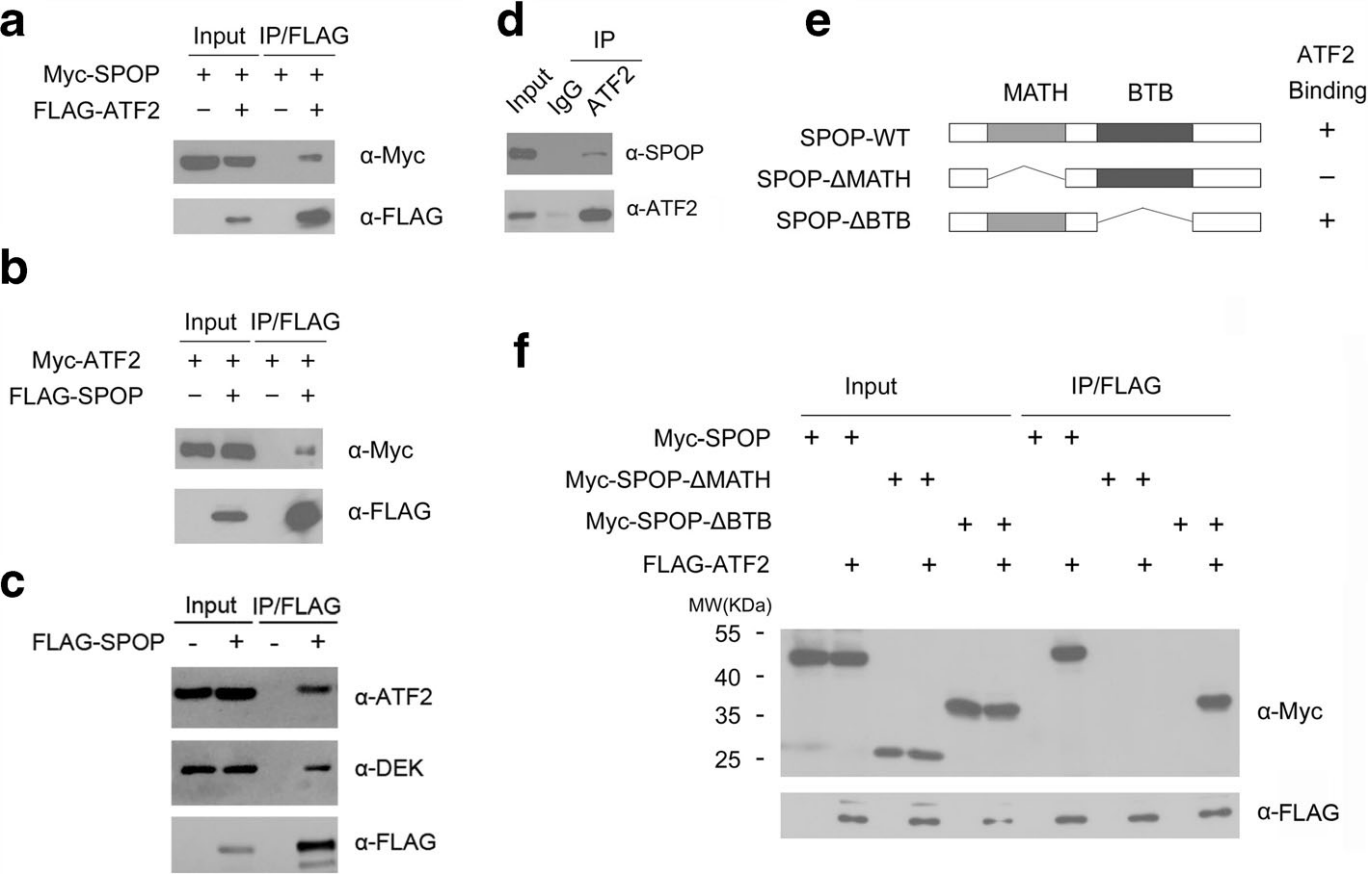
Identification of ATF2 as a novel SPOP Interactor. **a** and **b** Western blot of whole cell lysates(WCL) and co-IP samples of anti-FLAG antibody obtained from 293 T cells transfected with indicated plasmids. **c** Western blot of WCL and co-IP samples of anti-FLAG antibody obtained from C4–2 cells infected with lentivirus expressing FLAG-SPOP or control. The cells were treated with 20 μM MG132 for 8 h before harvesting. **d** Western blot of co-IP samples of IgG or anti-ATF2 antibodies obtained from cell lysates of C4–2 cells. The cells were treated with 20 μM MG132 for 8 h before harvesting. **e** Schematic representation of SPOP deletion mutants. Binding capacity of SPOP to ATF2 is indicated with the symbol. **f** Western blot of WCL and co-IP samples of anti-FLAG antibody obtained from 293 T cells transfected with indicated plasmids

To verify that ATF2 is a bona fide SPOP interacting protein, we first examined whether SPOP could interact with ATF2 in cells. We co-expressed FLAG-ATF2 and Myc-SPOP constructs in 293 T cells and then performed co-immunoprecipitation (co-IP) analysis with the anti-FLAG antibody. As shown in Fig. 1a, Myc-SPOP was successfully co-immunoprecipitated by FLAG-ATF2, suggesting an interaction between the two exogenously expressed proteins. Similar results were obtained in additional co-IP experiments, in which FLAG-SPOP was able to co-immunoprecipitate Myc-ATF2 (Fig. 1b). FLAG-SPOP was able to immunoprecipitate endogenous ATF2, and a known SPOP substrate DEK in C4–2 cells (Fig. 1c). Next, we investigated the potential binding between endogenous SPOP and ATF2. We performed immunoprecipitation using the anti-ATF2 antibody in cell lysates prepared from C4–2 cells. As shown in Fig. 1d, endogenous SPOP was efficiently co-immunoprecipitated by the ATF2 antibody, suggesting an endogenous interaction between these two proteins.

SPOP contains two structural domains: a substrate-binding MATH domain at the N-terminus and a CUL3-binding BTB domain at the C-terminus. To determine the domain that mediates its interaction with ATF2, we generated two deletion mutants of SPOP corresponding to the deletion of these two domains: SPOP-ΔBTB and ΔMATH (Fig. 1e). Co-IP assay was then performed to test the ability of overexpressed ATF2 to bind the two deletion mutants in 293 T cells. As shown in Fig. 1f, while full-length SPOP (SPOP-WT) and SPOP-ΔBTB efficiently interacted with ATF2, the interaction was abolished between SPOP-ΔMATH and ATF2. Taken together, these findings demonstrate that SPOP interacts with ATF2 in vivo through the MATH domain.

### ATF2 is a bona fide substrate of the SPOP-CUL3-RBX1 E3 ubiquitin ligase complex

**Fig. 2:**
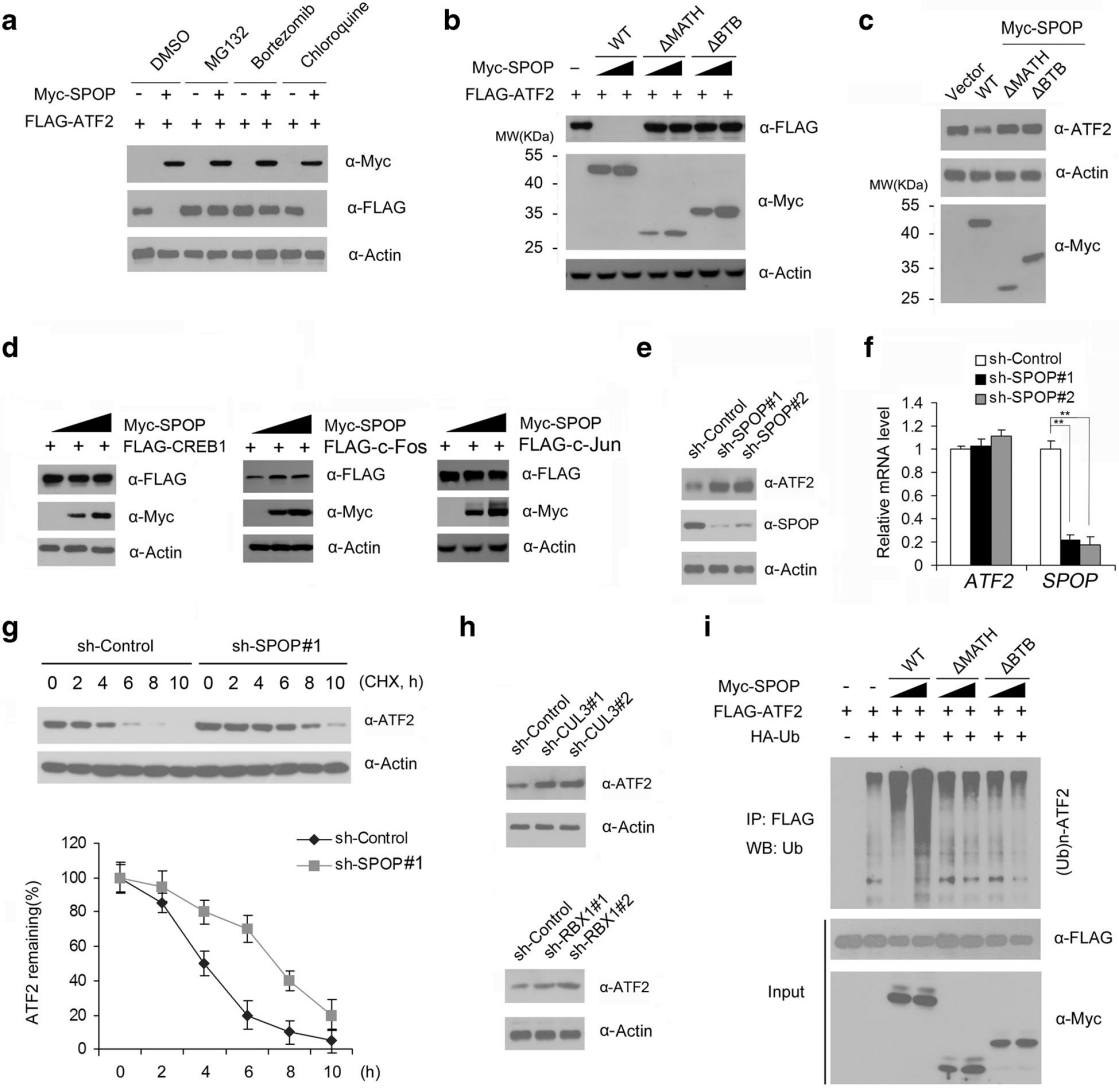
ATF2 is a bona fide substrate of the SPOP-CUL3-RBX1 E3 ubiquitin ligase complex. **a** Western blot of WCL from 293 T cells transfected with the indicated plasmids. and treated with MG132 (20 μM), Bortezomib (200 nM), Chloroquine (100 mM) or DMSO for 8 h. Actin was used as a loading control. **b** Western blot of WCL of 293 T cells transfected with indicated plasmids. **c** Western blot of WCL of C4–2 cells transfected with indicated plasmids. **d** Western blot of WCL of 293 T cells transfected with indicated plasmids. **e** Western blot of the WCL of C4–2 infected with control or lentivirus expressing SPOP-specific shRNAs (shSPOP#1,2). **f** Quantitative RT-PCR measurement of *SPOP* and *AT*F2 mRNA levels in SPOP-depleted C4–2 cells. GAPDH mRNA levels were used for normalization. Standard deviation (S.D.) of at least three independent experiments is shown to indicate statistical significance. **p<0.01. **g** Western blot of WCL of C4–2 cells infected with control or lentivirus expressing SPOP-specific shRNAs and then treated with 50 μg/ml cycloheximide (CHX) and harvested at different time points (upper panel). At each time point, the intensity of each BET protein was normalized to the intensity of actin and then to the value at 0 h (lower panel). **h** Western blot of the WCL of C4–2 cells infected with control or lentivirus expressing CUL3 or RBX1-specific shRNAs (sh-CUL3#1,2; sh-RBX1#1,2). **i** Western blots of the products of in vivo ubiquitination assays performed using cell lysate from 293 T cells transfected with the indicated plasmids and treated with 20 μM MG132 for 8 h

We next explored whether the SPOP-CUL3-RBX1 E3 ubiquitin ligase complex could promote the ubiquitination and degradation of ATF2. As shown in Fig. 2a, expression of SPOP decreased the levels of ectopically co-expressed ATF2 protein in a dose-dependent manner. This effect was completely blocked when cells were treated with the proteasome inhibitors MG132 or Bortezomib. In contrast, the lysosome inhibitor chloroquine had no impact on SPOP-mediated ATF2 degradation. These results indicated that SPOP downregulates ATF2 protein via the proteasomal, but not the lysosomal, degradation pathway. Moreover, SPOP-WT, but not the SPOP-ΔBTB or SPOP-ΔMATH mutant, promoted ATF2 degradation (Fig. 2b), indicating that the BTB and MATH domains are both required for SPOP-mediated ATF2 degradation. This result is consistent with the C-terminal BTB domain of SPOP interacting with the scaffold protein CUL3 [32] and the N-terminal MATH domain interacting with ATF2 (Fig. 1f). Similarly, overexpression of SPOP-WT but not the SPOP-ΔBTB or ΔMATH mutant resulted in a modest reduction of endogenous ATF2 protein in C4–2 cells (Fig. 2c). We also explored whether other members of the ATF/CREB bZIP family, including CREB1, c-Fos and c-Jun, were downregulated by SPOP, and the results showed that these proteins was not targeted by SPOP for downregulation (Fig. 2d).

We also depleted endogenous SPOP in C4–2 cells using two SPOP-specific shRNAs and observed an increase in ATF2 protein levels (Fig. 2e). To exclude the possibility that ATF2 protein elevation resulted from transcriptional upregulation, we performed qRT-PCR to measure the mRNA levels of ATF2 in SPOP-depleted C4–2 cells. In contrast to the significant decrease of SPOP mRNA levels in SPOP-depleted cells, ATF2 mRNA levels were similar to those in control cells (Fig. 2f), indicating that the effect of SPOP on ATF2 is not mediated through the regulation of ATF2 mRNA expression. Moreover, knockdown of SPOP remarkably prolonged the half-life of endogenous ATF2 protein in C4–2 cells (Fig. 2g), further suggesting that SPOP regulates ATF2 at the protein level.

We next evaluated whether other subunits of the SPOP-CUL3-RBX1 E3 ubiquitin ligase complex are also required for ATF2 degradation. We depleted RBX1 or CUL3 by two gene-specific shRNAs and examined the changes in ATF2 protein level in C4–2 cells. As shown in Fig. 2h, knockdown of either RBX1 or CUL3 resulted in a markedly increase in ATF2 protein levels, suggesting that the SPOP-E3 ubiquitin ligase complex is required for the degradation of ATF2. To further determine whether ATF2 was degraded through SPOP-mediated polyubiquitination, HA-Ubiquitin and Flag-ATF2 constructs were co-expressed in 293 T cells with different levels of SPOP-WT, SPOP-ΔBTB or ΔMATH mutant. As shown in Fig. 2i, ATF2 protein was robustly polyubiquitinated upon co-expression of SPOP-WT in a dose-dependent manner. In contrast, little or no ATF2 polyubiquitination was observed in SPOP-ΔBTB or ΔMATH expressing cells (Fig. 2i). Taken together, these data demonstrate that the SPOP-CUL3-RBX1 E3 ubiquitin ligase complex regulates ATF2 protein stability through ubiquitin-dependent proteasomal degradation in prostate cancer cells.

### Multiple Ser/Thr (S/T)-rich motifs in ATF2 are required for SPOP-mediated ATF2 degradation

**Fig. 3:**
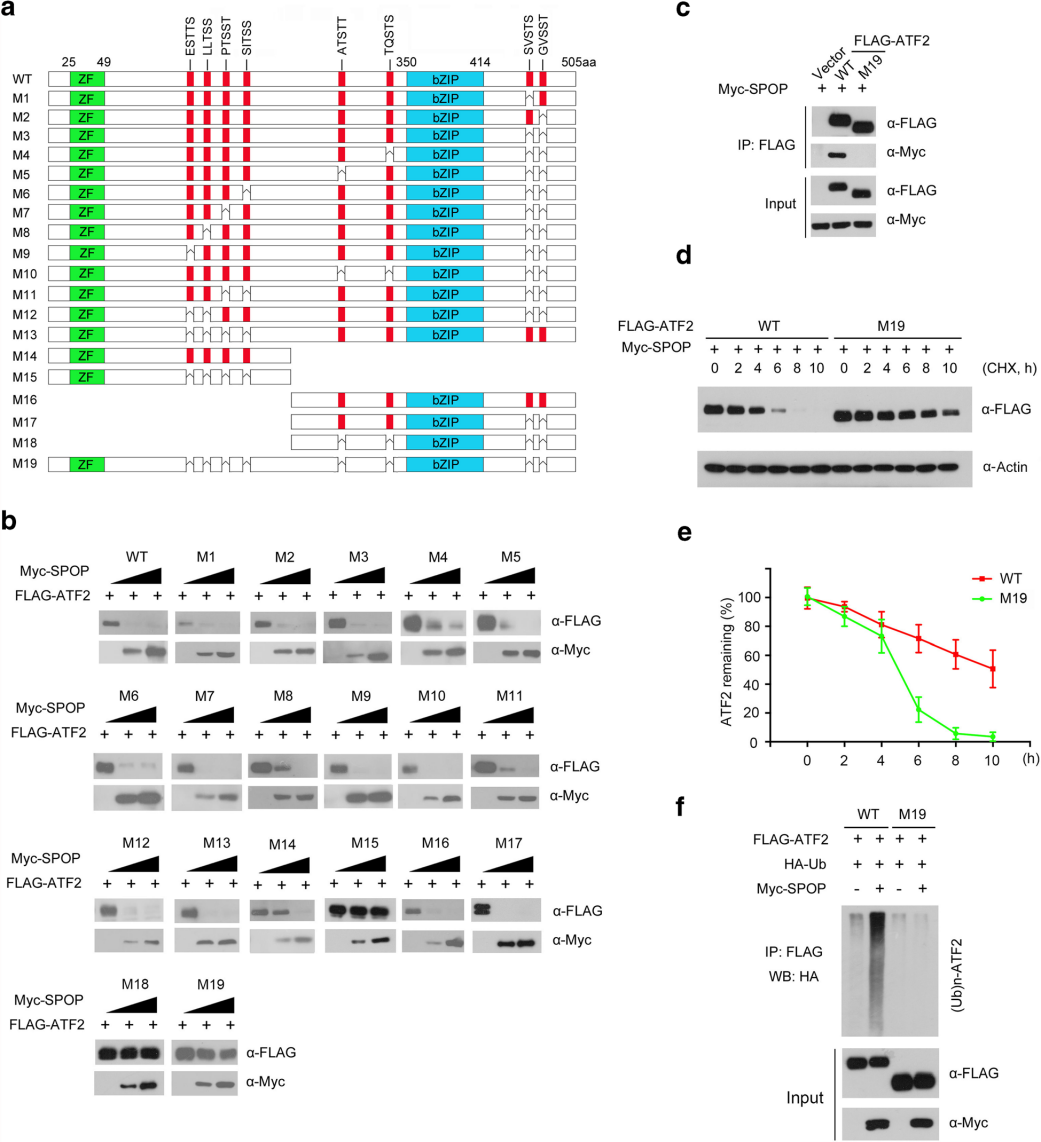
Multiple Ser/Thr (S/T)-rich motifs in ATF2 are required for SPOP-mediated ATF2 degradation. **a** Diagram showing wild-type ATF2 proteins and S/T rich motif-deleted mutants. The S/T rich motif is depicted in red. **b** Western blot of WCL of 293 T cells transfected with indicated plasmids. **c** Western blot of WCL and co-IP samples of anti-FLAG antibody obtained from 293 T cells transfected with indicated plasmids. **d** and **e** Western blots of WCL from 293 T cells transfected with the indicated constructs, treated with 50 μg/ml cycloheximide (CHX) and harvested at different time points. **e** Quantification of the western blots carried out in **d**. At each time point, the intensity of ATF2 protein was normalized to the intensity of actin and then to the value at 0 h. **f** Western blots of the products of in vivo ubiquitination assays performed using cell lysate from 293 T cells transfected with the indicated plasmids and treated with 20 μM MG132 for 8 h

Previous studies reported that one or several SBC motifs (Φ-π-S-S/T-S/T; Φ: nonpolar residues, π: polar residues) are present in known SPOP substrates [8]. We examined the protein sequence of ATF2 that is required for SPOP-binding. ATF2 contains two perfect matched SBC motif (192-PTSST-196 aa, 318-ATSTT-323 aa). Unexpectedly, deletion of either PTSST or ATSTT did not abolish SPOP-mediated ATF2 degradation (Fig. 3a and b). We seek the sub-optimal SBC motifs in ATF2 protein that contains three to four contiguous Ser/Thr (S/T) residues. Eight S/T-rich motifs resembling the SPOP-binding pattern dispersed in ATF2 protein sequence (Fig. 3a). To examine whether which S/T-rich motif (s) is required for SPOP-mediated ATF2 degradation, we generated eighteen ATF2 mutants (M1 to M18), in which the S/T-rich motifs were deleted independently or in combination (Fig. 3a). An ATF2-M19 mutant was also generated in which all eight S/T-rich motifs were deleted (Fig. 3a). We co-transfected 293 T cells with SPOP and wild-type ATF2 or one of the mutants. As shown in Fig. 3b, most mutants were still degraded by SPOP. Notably, ATF2 mutants with no S/T-rich motifs (ATF2-M15, M18 or M19) were resistant to SPOP-mediated degradation (Fig. 3b). Furthermore, the ATF2-M19 mutant lost the ability to bind SPOP (Fig. 3c). To further explore whether these S/T-rich motifs are important for ATF2 turnover, we measured the half-lives of ATF2-WT or the ATF2-M19 mutant in 293 T cells. As shown in Fig. 3d and e, the ATF2-M19 mutant exhibited a significantly prolonged half-life compared with ATF2-WT. To further determine the importance of these motifs as degrons, we performed in vivo ubiquitination assays in 293 T cells transfected with ATF2-WT or M19 with or without SPOP. The results showed that deletion of all S/T-rich motifs (ATF2-M19) totally abolished SPOP-mediated ATF2 ubiquitination (Fig. 3f).

The majority of known SPOP substrates contain 1–2 degrons. However, a few SPOP substrates, such as Ci or Gli3, contain multiple Ser/Thr-rich degrons like ATF2. Previous study demonstrated multiple weak linear motifs cooperatively enhance recruitment and processivity in SPOP-Mediated Gli3 ubiquitination [33]. Taken together, these observations demonstrate that multiple S/T-rich motifs functions as ATF2 degrons that are essential for SPOP binding and subsequent ubiquitination and degradation of ATF2.

### Prostate cancer-associated mutants of SPOP are defective in promoting ATF2 degradation and ubiquitination

**Fig. 4:**
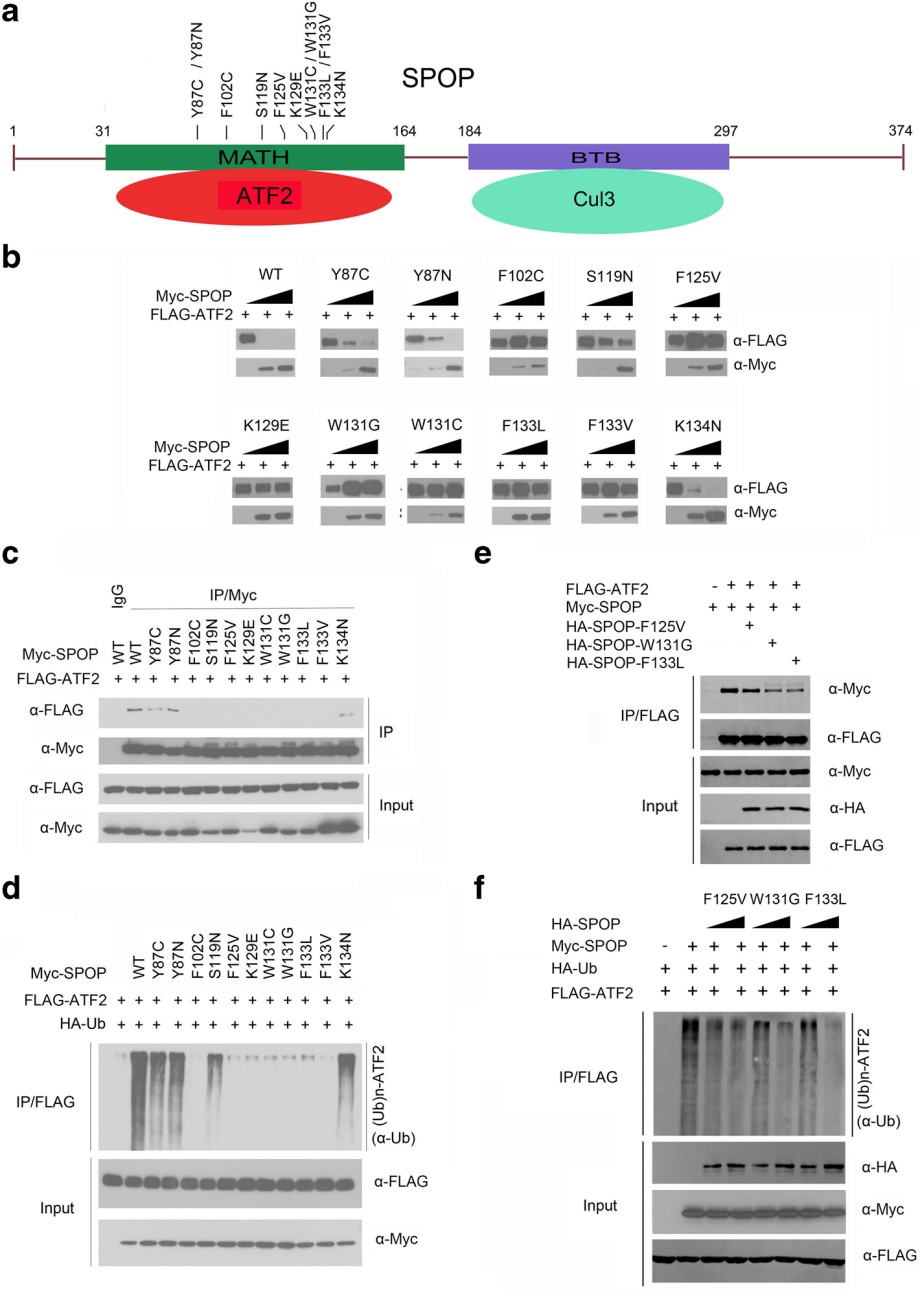
Prostate cancer-associated mutants of SPOP are defective in promoting ATF2 degradation and ubiquitination. **a** Distribution of the most common mutations in the SPOP gene found in prostate cancer. **b** Western blot of WCL of 293 T cells transfected with indicated plasmids. **c** Western blot of WCL and co-IP samples of anti-FLAG antibody obtained from 293 T cells transfected with indicated plasmids. **d** Western blots of the products of in vivo ubiquitination assays performed using cell lysate from 293 T cells transfected with the indicated plasmids and treated with 20 μM MG132 for 8 h. **e** Western blot of WCL and co-immunoprecipitation of anti-FLAG antibody in lysates from 293 T cells transfected with the indicated plasmids. **f** Western blot of the in vivo ubiquitination assay in 293 T cells transfected with the indicated plasmids and treated with 20 μM MG132 for 8 h

A previous study showed that SPOP was mutated up to 15% of patients with prostate cancer [34]. Notably, all the SPOP mutations found in prostate cancers exclusively occur in the MATH domain, which is responsible for substrate binding (Fig. 4a). Therefore, we speculated that prostate cancer-associated SPOP mutations may cause dysfunctions in regulating ATF2 protein levels. To test this hypothesis, we generated a series of Myc-tagged prostate cancer-associated mutants of SPOP, including Y87C, Y87N, F102C, S119 N, F125 V, K129E, W131G, W131C, F133 L, F133 V and K134 N, and examined their capacity to promote ATF2 degradation. As shown in Fig. 4b, we found that a group of SPOP mutants (SPOP-Y87N, Y87C, S119 N and K134 N, named here as SPOP type I mutants) displayed moderately reduced capacity to promote ATF2 degradation compared with SPOP-WT, whereas the mutants either did not induce significant changes in ATF2 protein levels (type II: SPOP-K129E, W131C and F133 V) or resulted in increased ATF2 protein levels (type III: SPOP-F102C, F125 V, W131G and F133 L).

We then examined the interactions between these SPOP mutants and ATF2 using co-immunoprecipitation assays. Most of the SPOP mutants showed failure to interact with ATF2, with the exception of the type I mutants (SPOP-Y87C, Y87N, S119 N and K134 N), which displayed moderately reduced binding capacity compared with SPOP-WT (Fig. 4c). Furthermore, in vivo ubiquitination assays indicated that type II and type III SPOP mutants lost the capacity to promote ATF2 polyubiquitination, while type I SPOP mutants generated relatively less polyubiquitinated forms of ATF2 compared with SPOP-WT (Fig. 4d). Together these results indicate the prostate cancer-associated mutations in SPOP can be categorized into the three groups as follows: type I SPOP mutants retain partial ATF2 binding, ubiquitination and degradation capacity; type II SPOP mutants lose ATF2 binding, ubiquitination and degradation capacity; and type III SPOP mutants also lose ATF2 binding capacity and ATF2 ubiquitination, but stabilizes ectopically co-expressed ATF2, suggesting a possible “dominant-negative effect”.

In prostate cancer, only one allele of the *SPOP* gene is mutated, and SPOP mutants exert their tumor-promoting functions in a dominant-negative manner to inhibit wild-type SPOP [3]. We hypothesized that some prostate cancer-associated mutations in SPOP might disrupt the interaction between wild-type SPOP and ATF2. Indeed, we found that co-expression of SPOP mutants (F125 V, W131G or F133 L) reduced the interaction between wild-type SPOP and ATF2 (Fig. 4e). Moreover, co-expression of SPOP mutants suppressed wild-type SPOP-induced ATF2 ubiquitination (Fig. 4f). Together our findings demonstrate that at least some prostate cancer-associated mutants of SPOP are defective in regulating ATF2 protein. Moreover, substrate binding is a prerequisite for SPOP-mediated ATF2 ubiquitination and degradation.

### ATF2 is an important mediator of SPOP inactivation-induced cell proliferation, migration and invasion

**Fig. 5:**
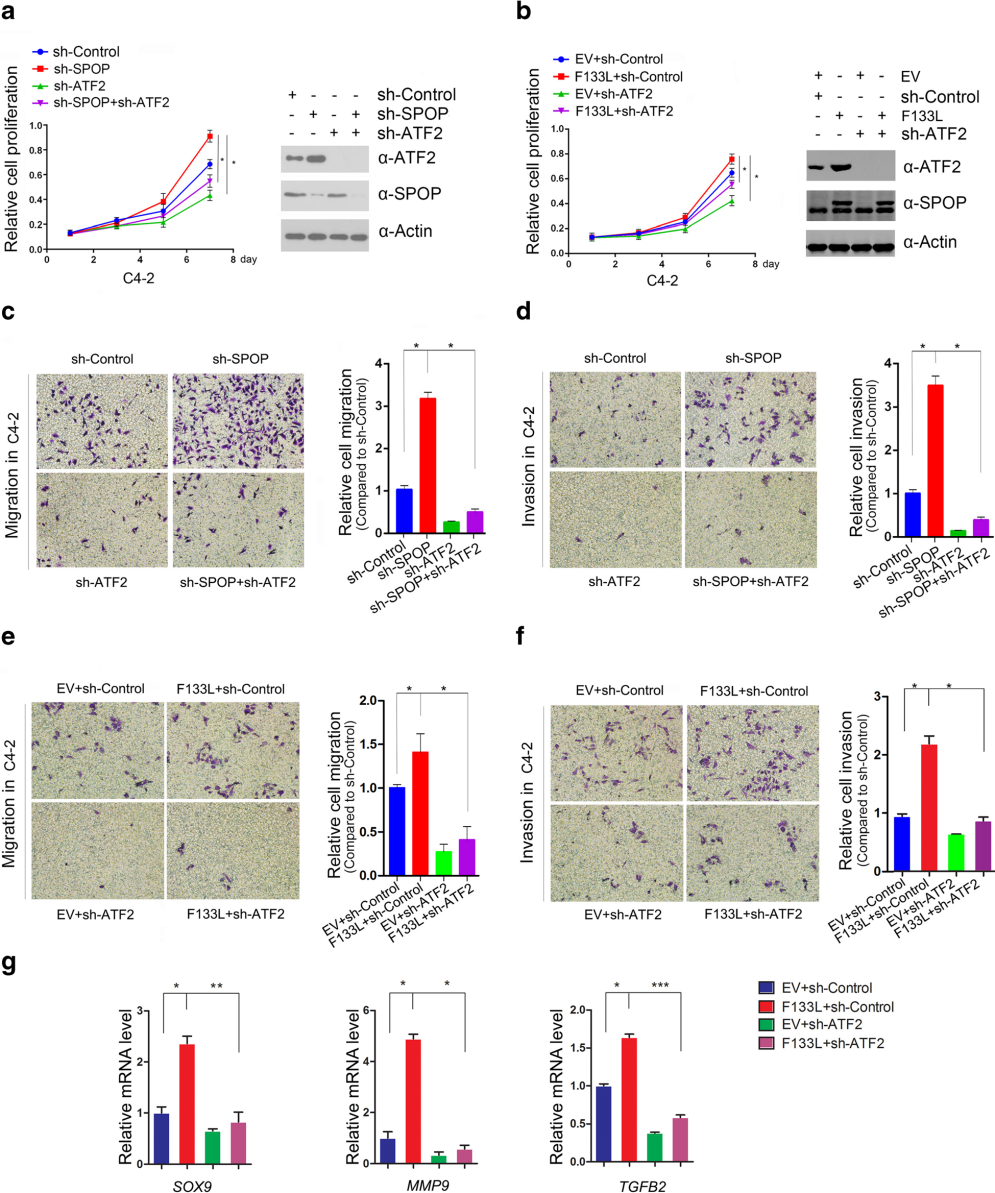
SPOP suppresses cell proliferation, invasion and migration partially dependent on ATF2. **a** Cell proliferation assay (left panel) and western blot (right panel) of C4–2 cells infected with lentivirus expressing the indicated shRNAs. All data shown are mean values ± SD (error bar) from three independent experiments. *p < 0.05 in C4–2 cells at day 7. **b** Assays shown in (A) were performed in C4–2 cells infected with empty vector or lentivirus expressing HA-SPOP-F133 V in combination with control shRNA or ATF2-spefic shRNAs. **c** and **d** Cell migration (C) and invasion (D) assay of C4–2 cells infected with lentivirus expressing the indicated shRNAs. All data shown are mean values ± SD from three replicates. *p < 0.05. **e** Cell migration and invasion **f** assay of C4–2 cells infected with lentivirus expressing HA-SPOP-F133 V in combination with control shRNA or ATF2-spefic shRNAs. All data shown are mean values ± SD from three replicates. *p < 0.05. **g** Quantitative RT-qPCR measurement of mRNA expression of three ATF2 transcriptional targets *SOX9*, *MMP9*, and *TGFB2* in C4–2 cells. *GAPDH* mRNA levels were used for normalization. Standard deviation (S.D.) of at least three independent experiments is shown to indicate statistical significance. *p<0.05,**p<0.01, **p<0.001

Previous studies reported that the expression of the prostate cancer-associated SPOP mutant or SPOP knockdown increases prostate cancer cell proliferation, migration and invasion [3, 12, 13]. Some studies have reported that ATF2 was associated with tumorigenesis and metastasis in several cancers [20, 24, 25, 28–30]. These observations led us to explore whether ATF2 is involved in SPOP mutants-mediated increase in cell proliferation, migration and invasion. Indeed, we found that depletion of ATF2 in C4–2 cells decreased cell proliferation (Fig. 5a). In contrast, depletion of SPOP enhanced cell proliferation, and co-depletion of SPOP and ATF2 reduced cell proliferation compared to levels with depletion of SPOP alone (Fig. 5a). More importantly, similar results were obtained when we overexpressed SPOP-F133 L mutant in cells with shRNA-mediated knockdown of endogenous SPOP (Fig. 5b). We also performed migration and invasion assays in C4–2 cells after depletion of SPOP or/and ATF2 (Fig. 5c and d). We observed the similar effects as the cell proliferation assay, in which the increase of migration and invasion in C4–2 cells by SPOP depletion was partly diminished by ATF2 depletion. Furthermore, SPOP-F133 L-expressing C4–2 cells showed similar results as cells with SPOP depletion by shRNA (Fig. 5e and f). Since ATF2 is a transcription factor that modulates some important genes involved in a variety of cellular processes, including cell proliferation, migration and invasion, we investigated whether SPOP mutation-caused ATF2 protein stabilization enhances ATF2 transcriptional outputs. As shown if Fig. 5g, stably overexpression of SPOP-F133 L mutant markedly increased the mRNA expression of ATF2 downstream target genes involved in cancer cell proliferation and invasion, including *SOX9*, *MMP9*, and *TGFB2*, [35–37], but this effect was largely abrogated by ATF2 depletion. Taken together, these results suggest that ATF2 is an important mediator of SPOP mutant-induced cell proliferation, migration and invasion.

### ATF2 protein levels were elevated in SPOP-MUT prostate cancer

**Fig. 6:**
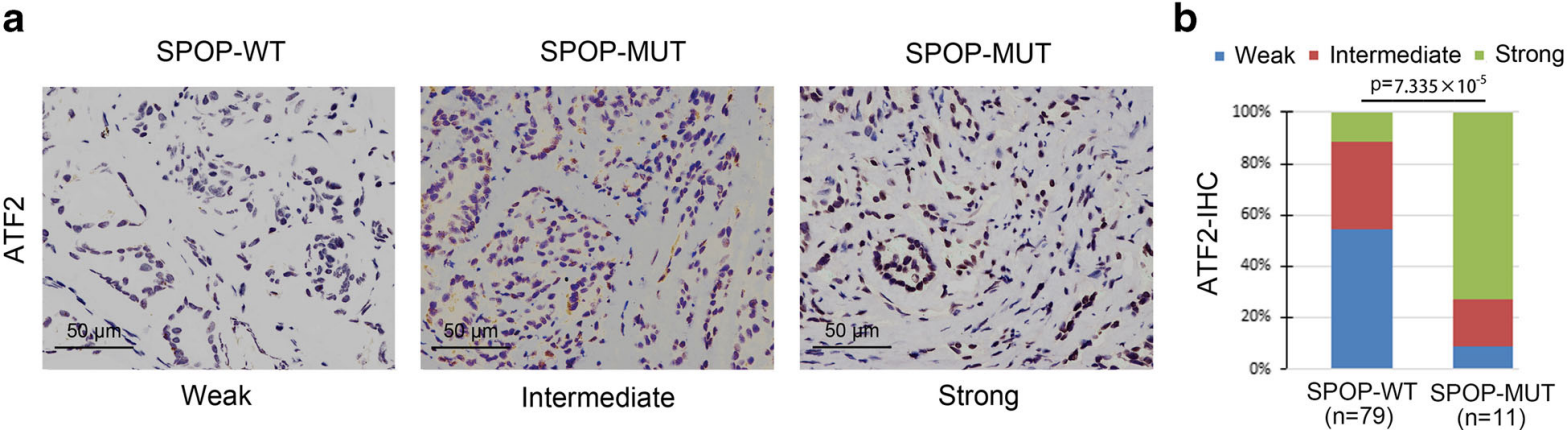
ATF2 protein expression is elevated in SPOP mutant-expressing prostate cancer specimens. **a** Representative images of ATF2 IHC from 90 cases of prostate cancer expressing wild-type SPOP or mutant SPOP (MUT). **b** Quantitative data of ATF2 protein staining in **a.** Statistical significance was determined by Wilcoxon rank-sum test

To examine the effect of SPOP mutations on ATF2 protein levels in specimens from individuals with prostate cancer, we analyzed ATF2 protein levels in a cohort for which a total of 90 primary prostate tumor samples were available (Additional file 1: Table S2). We identified 11 SPOP-mutated tumors through Sanger sequencing. The SPOP mutation frequency in our samples is consistent with previous findings in different cohorts of prostate cancer [3]. Immunohistochemistry (IHC) analysis showed that approximately 90% of SPOP-mutated tumors exhibited strong or intermediate staining for ATF2 (Fig. 6). In contrast, approximately 40% of tumors with wild-type SPOP exhibited strong or intermediate staining for ATF2 and the majority of the tumors with wild-type SPOP (approximately 60%) exhibited weak staining (Fig. 6). These findings indicate that SPOP mutations correlates with ATF2 protein levels in primary prostate cancer specimens.

## Discussion

Although SPOP mutation is now recognized as a distinct molecular feature in a subtype of prostate cancer, the functional impact of these mutations on prostate tumorigenesis and metastasis is still not fully understood [5]. Previous studies showed that SPOP inactivation increased cell proliferation, migration and invasion in prostate cancer cell lines. These effects were partly dependent on stabilization of SPOP substrates such as AR, ERG and BET proteins [9–14]. In this study, we demonstrated for the first time that ATF2 is a bona fide substrate for the SPOP-CUL3-RBX1 E3 ubiquitin ligase complex. We also revealed that some prostate cancer-associated SPOP mutants show impaired binding to ATF2 proteins, resulting in impaired proteasomal degradation and accumulation of ATF2 in prostate cancer cell lines and cancer specimens, which partly contributes to SPOP inactivation-induced prostate cancer cell migration and invasion.

Accumulating evidence supports the notion that ATF2 plays a critical role during prostate cancer initiation and progression. Analysis of samples from normal prostate tissue, benign prostatic hyperplasia and prostate cancer revealed that phosphorylated ATF2 is overexpressed in benign prostatic hyperplasia and, much higher, in prostate tumors [26]. These observations suggest that phosphorylated ATF2 enhances survival and cell proliferation, promoting prostate cancer progression. Another study showed that the expression of glucocorticoid receptors (GR) are decreased or absent in majority of prostate cancer samples. Reconstitution of GR expression in LNCaP cells resulted in decreased expression of MAP-kinases (MAPK) activity, subsequent downregulation of numerous transcription factors, including ATF2 [38]. Therefore, GR acts as a tumor suppressor to suppress prostate cancer. An oncogenic long non-coding RNA UCA1 positively regulated ATF2 expression through functioning as a competing endogenous RNA (ceRNA). Therefore, inhibition of UCA1 suppressed prostate cancer cells proliferation, migration and invasion [39]. These findings together with ours supported ATF2 act as an oncogene in prostate cancer, and its protein and mRNA level are dysregulated in prostate cancer. Development of therapeutic approaches for targeting aberrant ATF2 activation could be a viable treatment in prostate cancer.

## Conclusion

Taken together, the study provides novel insights into the aberrant regulation of ATF2 in prostate cancer, showing that ATF2 is an important mediator of SPOP inactivation-induced cell proliferation, migration and Invasion. This understanding may help with the development of potential therapeutic approaches for patients bearing SPOP mutations and cancer metastasis.

## Additional file

Additional file 1: **Table S1.** SPOP mutation status, ATF2 IHC scores in 90 cases of prostate cancer specimens and the associated clinical information. **Table S2.** Positive hits of the yeast two-hybrid screening. (XLSX 2194 kb)

## Abbreviations

AR: Androgen receptor
ATF2: Activating transcription factor 2
BTB: Bric-a-brac/Tramtrack/Broad complex
ceRNA: Competing endogenous RNA
CRLs: CULLIN-RING ligases
SPOP: Speckle-type POZ Protein
SRC-3: Steroid receptor coactivator 3
WCL: Whole cell lysates
